# Minimum effective concentration of ropivacaine for ultrasound-guided pericapsular nerve group block to relieve spinal anesthesia positioning pain in elderly patients with femoral intertrochanteric fracture: a double-blind biased-coin dose-finding study

**DOI:** 10.3389/fmed.2026.1924751

**Published:** 2026-08-07

**Authors:** Wenwen Zhai, Feng Yue, Yujie Wang, Mingfeng Mao, Fenglin Guo, Fang Guo, Bo Jiang, Min Li, Xiangyang Guo

**Affiliations:** 1 Department of Anesthesiology, Peking University Third Hospital, Beijing, China; 2 Executive Office, Center of Quality Control and Improvement on Clinical Anesthesia, Beijing, China; 3 Department of Otorhinolaryngology, The Sixth Medical Center of Chinese PLA General Hospital, Beijing, China; 4 Department of Radiology, The Seventh Medical Center of Chinese PLA General Hospital, Beijing, China

**Keywords:** biased-coin up-down design, femoral intertrochanteric fracture, pericapsular nerve group block, positioning pain, ropivacaine

## Abstract

**Background:**

The optimal ropivacaine concentration for ultrasound-guided pericapsular nerve group (PENG) block to relieve spinal anesthesia positioning pain in elderly patients with femoral intertrochanteric fracture remains unclear. We aimed to estimate the minimum effective concentration (MEC) and explore concentration-related motor impairment.

**Methods:**

In this double-blind biased-coin up-and-down study, elderly patients scheduled for spinal anesthesia underwent ultrasound-guided PENG block with 20 mL ropivacaine. The initial concentration was 0.10%. For a successful block, the subsequent patient was assigned to the same concentration (probability = 0.89) or a 0.05% lower concentration (probability = 0.11). For a failed block, the concentration was increased by 0.05% for the next patient. The trial was terminated when 45 successful blocks were achieved. Block success was defined as a pain score ≤ 3 at rest and during passive leg raise 30 min after block. The primary outcome was MEC90 estimated by isotonic regression. Secondary outcomes included MEC50, concentration-specific success rates, quadriceps weakness, and onset time of analgesia.

**Results:**

Fifty-two patients completed the sequential trial. Isotonic regression estimated MEC90 at 0.25% (95% CI, 0.20%−0.30%) and MEC50 at 0.20% (95% CI, 0.20%−0.20%). Success rates were 0% at 0.10% and 0.15%, 89.5% at 0.20%, 96.0% at 0.25%, and 100% at 0.30%. Quadriceps weakness at 6 h increased with concentration, occurring in 5.3% at 0.20%, 36.0% at 0.25%, and 50.0% at 0.30% (trend *P* = 0.007; risk ratio comparing high- with low-concentration groups, 8.72; 95% CI, 1.21–62.72). This safety comparison was exploratory. No local anesthetic systemic toxicity or serious adverse events were observed.

**Conclusions:**

In elderly patients with femoral intertrochanteric fracture receiving ultrasound-guided PENG block with a fixed 20 mL volume of ropivacaine, the prespecified isotonic regression-estimated MEC90 for relieving spinal anesthesia positioning pain was 0.25%. Exploratory safety analyses suggested that quadriceps weakness at 6 h was more common at concentrations ≥0.25%, but these analyses were underpowered and hypothesis-generating. Future studies should determine whether modifying volume, concentration, or total dose can preserve analgesia while reducing motor impairment.

**Clinical trial registration:**

identifier ChiCTR2500102032.

## 1 Introduction

Hip fractures represent a substantial healthcare burden among older adults and are typically accompanied by severe pain at rest and during movement (1, 2). Adequate pain control prior to spinal anesthesia is clinically critical for this patient population, as positioning-induced pain can hinder successful neuraxial block placement, amplify sympathetic stress responses, and increase the need for rescue systemic opioids (3, 4). Spinal anesthesia is widely adopted for hip fracture repair and has been associated with selected perioperative advantages, such as shortened hospital length of stay, reduced intraoperative blood loss, and lower risks of certain pulmonary and thromboembolic complications in observational research (5). Nevertheless, high-quality randomized controlled trials including REGAIN and RAGA have failed to identify significant differences in mortality or postoperative delirium between spinal and general anesthesia for elderly hip fracture patients (6, 7). Clinicians commonly rely on systemic opioids, femoral nerve block (FNB), or fascia iliaca compartment block (FICB) for preoperative pain relief (8–10), yet each approach carries notable drawbacks in older individuals: opioids trigger multiple adverse effects, while FNB and FICB frequently induce quadriceps weakness that impedes early mobilization and elevates fall risk (11–13). Beyond intraoperative anesthesia and acute pain management, contemporary literature highlights comprehensive perioperative care bundles, including risk stratification, enhanced rehabilitation protocols, and bone metabolism-targeted adjuvant therapies, to facilitate postoperative recovery in patients with intertrochanteric fractures (14, 15).

The pericapsular nerve group (PENG) block, first described in 2018, is a relatively recent regional analgesic technique targeting articular branches of the femoral, obturator, and accessory obturator nerves that innervate the pain-sensitive anterior hip capsule (16). This approach is intended to target sensory articular branches and may relieve rest and movement-evoked hip pain while preserving motor function; however, unintended local anesthetic spread to adjacent femoral motor branches may occur in clinical practice. A uniform 20 mL volume of local anesthetic is widely adopted in anatomical and clinical PENG studies to achieve adequate spread around target sensory branches (17). Published studies have used ropivacaine concentrations ranging from 0.2 to 0.75% combined with 15–20 mL injection volume for PENG block, but no consensus exists regarding the optimal concentration (18). High local anesthetic concentrations raise two clinical concerns: elevated risks of local anesthetic systemic toxicity, and quadriceps weakness that increases fall risk (19). In fixed-volume protocols, increasing concentration also increases the total ropivacaine dose, making it difficult to distinguish concentration-related from total-dose-related motor effects. Although PENG block is increasingly applied for preoperative hip fracture pain control, to our knowledge, no prospective dose-finding trial has quantified the minimum effective ropivacaine concentration to achieve satisfactory positioning analgesia, nor systematically explored concentration-associated quadriceps weakness among elderly patients with intertrochanteric fractures.

Therefore, this study's primary objective was to estimate the MEC90 of ropivacaine for ultrasound-guided PENG block with a standardized 20 mL volume, to alleviate positioning pain prior to spinal anesthesia in elderly patients with femoral intertrochanteric fractures. Our secondary objectives included descriptive comparisons of analgesic efficacy across tested concentrations and exploratory safety assessments focusing on quadriceps weakness.

## 2 Methods

### 2.1 Ethical approval and trial registration

This study was approved by the Ethics Committee of Peking University Third Hospital (Approval No.: M20250103). The trial was prospectively registered with the Chinese Clinical Trial Registry on May 7, 2025, with the registration number ChiCTR2500102032. All procedures were conducted in accordance with the Declaration of Helsinki (2013 version) and relevant national medical research regulations. The study was reported in accordance with the CONSORT statement extension for dose-finding designs.

### 2.2 Patient recruitment

Elderly patients (aged ≥ 65 years) with American Society of Anesthesiologists (ASA) physical status I–III, who were scheduled for surgical treatment of femoral intertrochanteric fracture under spinal anesthesia, were recruited from a single center between June 1 and December 31, 2025. Written informed consent was obtained from all participants or their legal guardians for patients with mild cognitive impairment unable to fully comprehend the trial protocol, and this consent explicitly granted permission to publish de-identified ultrasound anatomical images in peer-reviewed journals; all figures have been fully de-identified with all recognizable personal features removed to protect patient confidentiality.

Patients were excluded if they met any of the following criteria: (1) Coagulation disorders (e.g., abnormal prothrombin time, activated partial thromboplastin time, or platelet count < 100 × 10^9^/L) or a history of open lumbar surgery; (2) Local infection, inflammation, or skin lesions at the PENG block puncture site; (3) Inability to cooperate due to hearing impairment, aphasia, severe mental disorders, or other cognitive deficits; (4) Pre-existing lower extremity neuromuscular disorders (e.g., peripheral neuropathy, myasthenia gravis) or a history of lower extremity nerve injury; (5) Known allergy to local anesthetics (including ropivacaine or lidocaine); (6) Refusal to participate in the trial or inability to complete follow-up assessments.

### 2.3 Trial design

This study was designed as a double-blind, sequential dose-finding trial to estimate the MEC90 of ropivacaine for ultrasound-guided single-injection PENG block in relieving positioning pain during spinal anesthesia. A biased-coin up-and-down (BCD) design was adopted, in which the concentration for each patient was sequentially determined based on the previous patient's response. The initial concentration was 0.10%. For successful blocks, the next patient received either the same concentration (probability = 0.89) or a 0.05% lower concentration (probability = 0.11); for failures, concentration increased by 0.05%. To minimize the risk of local anesthetic systemic toxicity, the maximum ropivacaine concentration was set at 0.50%; if a patient receiving this maximum concentration still had block failure, the next patient continued to receive 0.50% ropivacaine (no further concentration increase). The choice of a 0.89/0.11 transition probability follows the standard BCD framework proposed by Durham and Flournoy for targeting the 90th percentile of the dose–response curve (20).

To preserve allocation concealment in this sequential dose-finding design, the biased-coin decision sequence was generated before trial initiation by an independent statistician who was not involved in patient recruitment, block performance, outcome assessment, or data analysis. The sequence was generated in R version 4.4.3 and applied only after a successful block, when the next patient was allocated either to the same ropivacaine concentration with a probability of 0.89 or to the next lower concentration by one 0.05% step with a probability of 0.11. After a failed block, the next concentration was increased by one 0.05% step according to the prespecified BCD algorithm, without use of the biased-coin sequence.

The biased-coin decision sequence was kept by the hospital pharmacy in sequentially numbered, opaque, tamper-evident envelopes. After the 30-min block outcome of each patient had been recorded by a blinded assessor, an independent investigator who was not involved in outcome assessment determined the concentration for the next patient according to the prespecified algorithm. When the preceding block was successful, the investigator opened the next envelope to obtain the biased-coin decision; when the preceding block failed, the concentration was increased according to protocol. The assigned study solution was then prepared by pharmacy personnel in a 20 mL syringe. All syringes were identical in appearance and volume. Patients, the anesthesiologist performing the block, surgeons, postoperative caregivers, outcome assessors, and the data analyst remained blinded to the assigned concentration until database lock and completion of the primary analysis.

### 2.4 PENG block

All patients received premedication with intravenous midazolam (0.01 mg/kg) in the pre-anesthesia preparation room, where noninvasive blood pressure monitoring, pulse oximetry, and electrocardiogram (ECG) were continuously performed to monitor vital signs. A single experienced anesthesiologist (with more than 5 years of experience in ultrasound-guided regional nerve block) performed all PENG block procedures 30 min before spinal anesthesia to ensure operational consistency.

**Figure 1:**
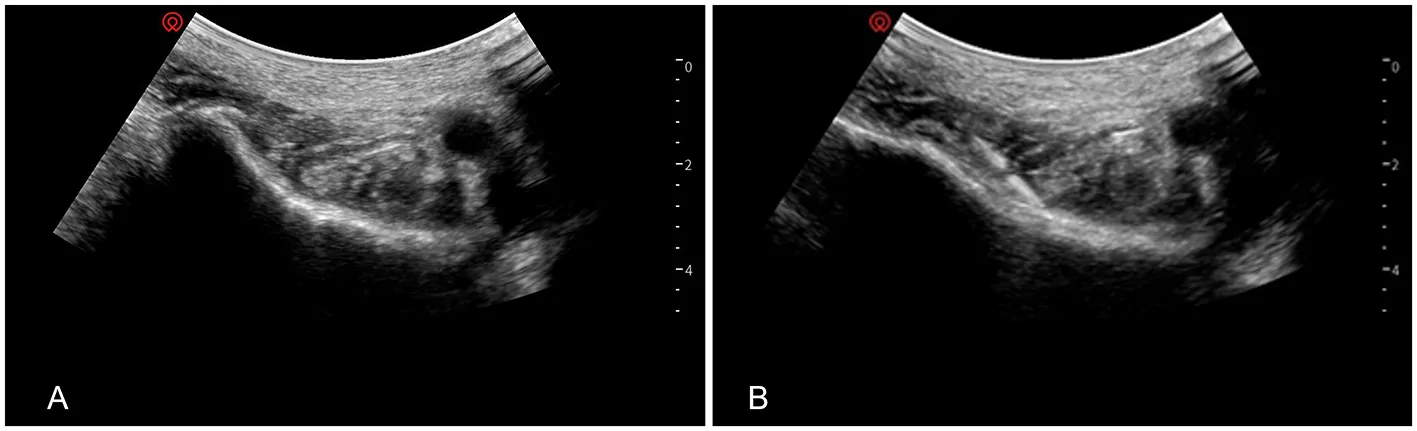
Ultrasound-guided pericapsular nerve group (PENG) block. **(A)** Pre-injection ultrasound image identifying the key anatomical landmarks. **(B)** Post-injection ultrasound image showing the spread deep to the psoas tendon over the superior pubic ramus.

With the patient in the supine position, the puncture area was disinfected with iodophor and draped in a sterile manner. Subcutaneous infiltration anesthesia was performed with 1 mL of 2% lidocaine to reduce puncture pain. A high-frequency linear-array ultrasonic transducer (TE9S, Mindray, Shenzhen, China) was placed parallel to the inguinal crease at the level of the anterior superior iliac spine (ASIS), and the probe was moved gradually caudally to obtain a clear ultrasound image. After identifying the anterior inferior iliac spine (AIIS), the probe was rotated slightly medially until the hyperechoic continuous shadow of the superior pubic ramus was visualized (Figure 1A). The psoas muscle with its prominent tendon was then identified just above the pubic ramus, and the target injection plane was the potential space between the psoas tendon and the superior pubic ramus.

After aligning the superior pubic ramus in the center of the ultrasound image and targeting the area just medial to the AIIS, a standard 21 G 100 mm block needle (Obcoo, Jinan, China) was inserted using the ultrasound-guided in-plane technique. Once the needle tip reached the target plane, and after negative aspiration to exclude intravascular placement, 20 mL of ropivacaine was injected at a constant rate of 5 mL/min. Real-time sub-tendinous injectate spread was verified for every participant under ultrasound (Figure 1B). If accidental intravascular injection was suspected (e.g., blood aspiration or sudden vital sign changes), the injection was immediately stopped, and the procedure was repeated after repositioning the needle.

### 2.5 Pain assessment and block success definition

Before the PENG block, all patients were instructed on the use of the numerical rating scale (NRS) for pain assessment during the pre-anesthetic visit. After PENG block administration, NRS scores at rest and during passive leg raise (PLR) were assessed every 5 min for 30 min by an outcome assessor blinded to the assigned concentration; the scores recorded at 30 min after block completion were used to define the primary outcome of block success. The patient was then transferred to the operating room, where standard monitoring (pulse oximetry, noninvasive blood pressure, and ECG) was reinitiated. The PLR maneuver was performed with the patient in the supine position by gently raising the affected limb to approximately 15° with slight hip flexion and full knee extension, mimicking the positioning required for spinal anesthesia. The PLR maneuver was performed in a standardized manner by trained research personnel, and patients were instructed not to actively contract the quadriceps during assessment.

Block success was defined as an NRS score ≤ 3 both at rest and during PLR 30 min after PENG block administration. If the NRS score was ≥ 4 at rest or during movement, the block was considered a failure, and rescue analgesia was provided with intravenous fentanyl (0.5–1 μg/kg) to ensure patient comfort during spinal anesthesia positioning. All enrolled patients, including those with block failure, proceeded to receive the planned spinal anesthesia at the L3–L4 interspace and were retained in the analysis. Onset time of analgesia in successful blocks was defined as the time from completion of local anesthetic injection to the first time point at which NRS scores were ≤ 3 both at rest and during PLR.

### 2.6 Spinal anesthesia procedure

After evaluating the PENG block effect, all patients underwent spinal anesthesia at the L3–L4 lumbar interspace with the affected limb in the dependent position. The following indicators were recorded during and after spinal anesthesia: (1) Need for rescue analgesia during spinal anesthesia positioning; (2) Ease of spinal positioning (EOSP), scored on a 4-point scale: 0 = Unable to assume the required position; 1 = Painful positioning requiring external assistance; 2 = Mild pain without the need for external assistance; 3 = Painless positioning; (3) Anesthesiologist satisfaction and patient satisfaction (both scored on a 5-point scale: 1 = Very dissatisfied, 5 = Very satisfied), after completion of spinal anesthesia positioning.

### 2.7 Outcome measures

Primary outcome: the primary estimand was the MEC90 of ropivacaine based on the binary block success response, which was defined as NRS ≤ 3 both at rest and during PLR 30 min after completion of the PENG block.

Secondary outcomes: (1) Need for rescue analgesia during spinal anesthesia positioning; (2) EOSP score; (3) Quadriceps weakness, defined as a score < 5 on the Oxford Muscle Strength Scale (0 = No visible or palpable muscle contractions; 1 = Flicker of contraction; 2 = Movement through full range with gravity eliminated; 3 = Movement through full range against gravity; 4 = Movement through full range against gravity and moderate resistance; 5 = Movement through full range against gravity and full resistance), was assessed at six and 24 h after PENG block by a blinded assessor; (4) PENG block performance time (from ultrasound probe placement to completion of local anesthetic injection); (5) Onset time of analgesia in successful blocks (as defined above); (6) Anesthesiologist and patient satisfaction scores; (7) Incidence of adverse events, including nausea and vomiting; local anesthetic systemic toxicity (manifested as central nervous system symptoms such as tinnitus, confusion, or convulsions, as well as cardiovascular symptoms including hypotension and bradycardia); clinically significant hypotension (defined as a systolic blood pressure < 90 mmHg or a ≥ 20% decrease from the baseline value); oxygen desaturation (SpO_2_ < 95%) occurring within 30 min after PENG block; and bleeding, hematoma at the puncture site, and infection.

### 2.8 Sample size calculation

Based on previous studies on BCD dose-finding trials, a minimum of 45 successful block responses is required to reliably estimate the MEC90 with acceptable precision (21, 22). According to simulations by Stylianou and Flournoy (23), 20–40 successful responses are typically sufficient to estimate the 90th percentile with reasonable bias and variance under a BCD design; we therefore conservatively set a target of 45 successful responses to enhance the precision of MEC90 estimation and accommodate the discrete concentration step size of 0.05%. Patients were prospectively enrolled sequentially until a total of 45 successful PENG blocks were achieved.

### 2.9 Statistical analysis

All statistical analyses were performed using R version 4.4.3 (R Foundation for Statistical Computing, Vienna, Austria). Continuous variables were assessed for normality using the Shapiro–Wilk test. Normally distributed variables are presented as mean ± standard deviation (SD) and were compared using the independent-samples *t*-test. Non-normally distributed variables are presented as median (interquartile range [IQR]) and were compared using the Wilcoxon rank-sum test. Categorical variables are presented as number (percentage) and were compared using Fisher's exact test. Given the sequential dose-finding design and the unbalanced sample sizes across concentration levels, between-group comparisons of baseline characteristics between success and failure groups are descriptive and exploratory rather than confirmatory.

The primary outcome was the MEC90 of ropivacaine for ultrasound-guided PENG block, and MEC50 was analyzed as a secondary concentration estimate. Concentration-response data were summarized by tested concentration level, and isotonic regression using the pooled-adjacent-violators algorithm (PAVA) was applied to obtain monotonic estimates of block success probability. MEC50 and MEC90 were defined as the lowest tested concentrations associated with isotonic estimated success probabilities of at least 50 and 90%, respectively. Bootstrap resampling with 10,000 iterations was used to estimate percentile-based 95% confidence intervals (CIs) for MEC50 and MEC90. All enrolled patients were included in the primary dose-finding analysis according to the concentration actually received. No imputation was performed because the binary block response was available for all participants.

Sensitivity analyses for the dose-response relationship were performed using generalized linear models with probit and logit links. Model-based estimates of MEC50 and MEC90 and their 95% CIs were derived from the fitted probit and logit models.

For adverse events, the incidence of quadriceps weakness was compared between the high-concentration group (≥0.25%) and low-concentration group (< 0.25%) using Fisher's exact test, and effect sizes were expressed as relative risk (RR) and odds ratio (OR) with 95% CIs. A Cochran–Armitage trend test was used to evaluate the concentration-dependent trend in quadriceps weakness across ordered concentration levels. In addition, exploratory logistic regression models were used to assess the association between increasing ropivacaine concentration and quadriceps weakness, with concentration entered as a continuous predictor scaled per 0.05% increase. A multivariable exploratory model further adjusted for age, sex, BMI, ASA physical status, and pre-block NRS score. The selection of the dichotomization threshold (0.25%) was prespecified based on the BCD-targeted MEC90 level and was intended to compare the clinically relevant high-vs. low-concentration ranges. Among patients with successful blocks, onset time of analgesia across effective concentration groups was compared using the Kruskal–Wallis test. A two-sided *P* value < 0.05 was considered statistically significant. Given the exploratory nature of secondary and safety analyses, no adjustment for multiple comparisons was performed, and these results should be interpreted as hypothesis-generating.

## 3 Results

### 3.1 Patient characteristics

**Figure 2:**
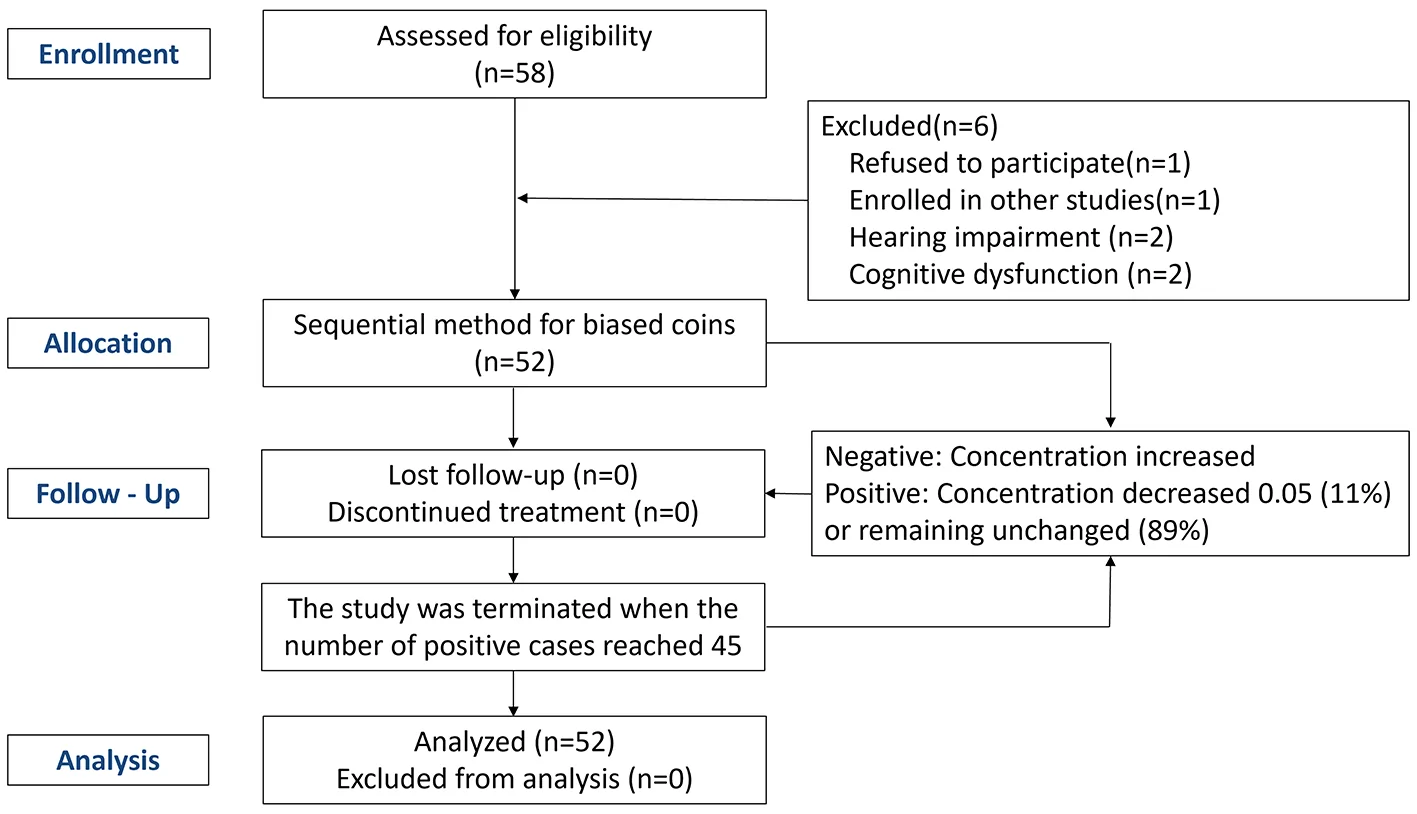
CONSORT flow diagram of patient enrollment, follow-up, and analysis.

Between June and December 2025, 52 patients were enrolled and completed the study protocol; no patient was excluded after enrollment (Figure 2). Baseline and procedural characteristics were summarized in Table 1. Although enrollment was open to patients with ASA physical status I–III, only patients with ASA II (*n* = 37) and III (*n* = 15) met the criteria during the study period; no ASA I patient was enrolled. As expected in a sequential dose-finding study, comparisons between successful and failed blocks were descriptive and exploratory; no apparent baseline imbalance was observed.

**Table 1:** Baseline characteristics and procedural data of the study patients.

Characteristics	Overall (*n* = 52)	Success (*n* = 45)	Failure (*n* = 7)	*P* value^a^
Age (years)	77 (67, 86)	77 (68, 86)	77 (65, 86)	0.798
Male sex	20 (38.5)	16 (35.6)	4 (57.1)	0.408
BMI (kg/m^2^)	24.0 ± 3.4	24.0 ± 3.4	23.5 ± 2.9	0.851
Weight (kg)	62 (60, 66)	62 (60, 65)	60 (59, 61)	0.664
Height (cm)	162 (155, 170)	160 (155, 170)	164 (155, 170)	0.466
ASA physical status				0.658
II	37 (71.2)	32 (71.1)	5 (71.4)	
III	15 (28.8)	13 (28.9)	2 (28.6)	
Block performance time (min)	11 (10, 12)	11 (10, 12)	11 (9, 13)	0.711
NRS before block	8 (8, 9)	8 (8, 9)	9 (8, 9)	0.221

^a^Comparisons between successful and failed blocks are exploratory only and should be interpreted cautiously because of the sequential dose-finding design and the small number of failed blocks.

Normally distributed continuous variables are presented as mean ± SD and were compared using the independent-samples *t*-test; non-normally distributed continuous variables are presented as median (IQR) and were compared using the Wilcoxon rank-sum test; categorical variables are presented as *n* (%) and were compared using Fisher's exact test.

BMI, body mass index; ASA, American Society of Anesthesiologists; NRS, numerical rating scale.

### 3.2 Primary outcome

**Figure 3:**
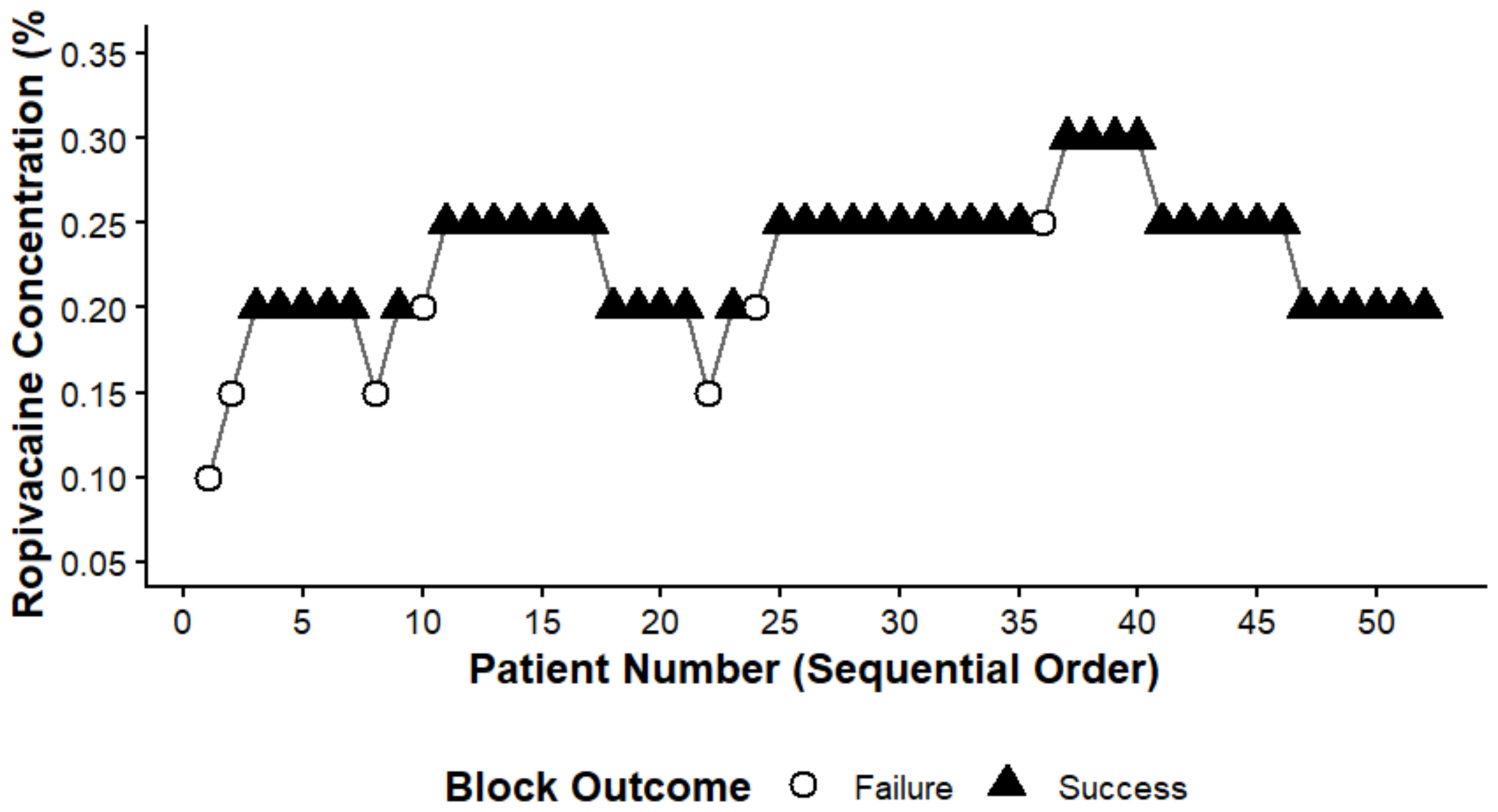
Sequential allocation of ropivacaine concentration and block outcomes in the biased-coin up-and-down design. Open circles indicate failed blocks, and filled triangles indicate successful blocks. Ropivacaine concentration was sequentially assigned according to the biased-coin up-and-down design, with tested concentrations ranging from 0.10% to 0.30% in 0.05% increments.

The sequential allocation of ropivacaine concentrations is shown in Figure 3. Test concentrations ranged from 0.10 to 0.30%, with a step size of 0.05%. The maximum prespecified concentration of 0.50% was not reached during the trial. Of the 52 enrolled patients, 45 (86.5%) achieved successful blocks and 7 (13.5%) experienced block failure.

Using isotonic regression with the pooled-adjacent-violators algorithm (PAVA), the MEC50 and MEC90 were estimated to be 0.20 and 0.25%, respectively. Bootstrap resampling with 10,000 iterations yielded percentile 95% CIs of 0.20%−0.20% for MEC50 and 0.20%−0.30% for MEC90 (Table 2 and Supplementary Figure S1).

**Table 2:** Primary concentration estimates and exploratory quadriceps weakness analyses.

Parameter	Estimate	95% CI	*P* value
Effective concentration estimates			
Isotonic regression MEC50	0.20%	0.20%−0.20%	–
Isotonic regression MEC90	0.25%	0.20%−0.30%	–
Probit model MEC50	0.169%	0.140%−0.198%	–
Probit model MEC90	0.217%	0.194%−0.239%	–
Logit model MEC50	0.173%	0.147%−0.198%	–
Logit model MEC90	0.212%	0.190%−0.233%	–
Analyses of quadriceps weakness at 6 h after PENG block			
High concentration group (≥0.25%) vs. low concentration group (< 0.25%), RR^a^	8.72	1.21–62.72	0.007^c^
High concentration group (≥0.25%) vs. low concentration group (< 0.25%), OR^a^	13.44	1.93–303.91	0.007^c^
Cochran–Armitage trend test across concentration levels	–	–	0.007
Logistic regression per 0.05% increase in concentration	4.81	1.62–20.80	0.013
Adjusted logistic regression per 0.05% increase in concentration^b^	5.56	1.73–26.50	0.011

^a^The risk ratio is presented as the preferred effect measure because quadriceps weakness was not rare; the odds ratio is provided for completeness.

^b^Adjusted for age, sex, BMI, ASA physical status, and pre-block NRS score.

^c^The same *P* value is shown for the RR and OR because both effect estimates refer to the same high- vs. low-concentration comparison; the *P* value was obtained using Fisher's exact test.

MEC values represent isotonic regression and model-based estimates; CIs were obtained via bootstrap for isotonic regression and model-based methods for probit and logit models.

MEC, minimum effective concentration; RR, relative risk; OR, odds ratio; CI, confidence interval.

**Figure 4:**
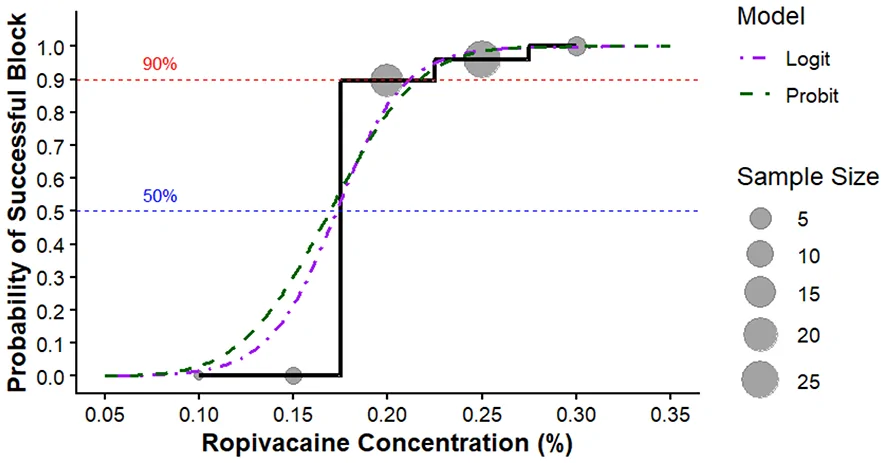
Dose-response relationship between ropivacaine concentration and probability of successful PENG block. The black step line represents the estimated probability of successful block obtained using isotonic regression with the pooled-adjacent-violators algorithm (PAVA). Gray circles indicate observed success rates at each tested concentration, with circle size proportional to sample size. The green dashed line and purple dot-dashed line represent the fitted probit and logit dose-response models, respectively. Horizontal dotted lines indicate the 50% and 90% probability thresholds.

In sensitivity analyses, the probit model estimated the MEC50 at 0.169% (95% CI, 0.140%−0.198%) and the MEC90 at 0.217% (95% CI, 0.194%−0.239%), whereas the logit model estimated the MEC50 at 0.173% (95% CI, 0.147%−0.198%) and the MEC90 at 0.212% (95% CI, 0.190%−0.233%) (Figure 4 and Table 2).

Concentration-specific success rates ranged from 0% at 0.10%−0.15% to 100% at 0.30%, with the greatest efficacy gain between 0.15 and 0.20% (Table 3).

**Table 3:** Concentration-specific efficacy and safety outcomes.

Ropivacaine concentration (%)	Patients, *n*	Successful blocks, *n* (%)	Isotonic estimated success probability	Rescue analgesia during positioning, *n* (%)	Quadriceps weakness at 6 h, *n* (%)	Oxford score distribution at 6 h, *n* (5/4/3/ ≤ 2)	Quadriceps weakness at 24 h, *n* (%)	Nausea/ vomiting, *n* (%)
0.10	1	0 (0.0)	0.000	1 (100.0)	0 (0.0)	1/0/0/0	0 (0.0)	0 (0.0)
0.15	3	0 (0.0)	0.000	3 (100.0)	0 (0.0)	3/0/0/0	0 (0.0)	0 (0.0)
0.20	19	17 (89.5)	0.895	2 (10.5)	1 (5.3)	18/1/0/0	0 (0.0)	1 (5.3)
0.25	25	24 (96.0)	0.960	1 (4.0)	9 (36.0)	16/8/1/0	0 (0.0)	0 (0.0)
0.30	4	4 (100.0)	1.000	0 (0.0)	2 (50.0)	2/1/1/0	0 (0.0)	1 (25.0)
Overall	52	45 (86.5)	—	7 (13.5)	12 (23.1)	40/10/2/0	0 (0.0)	2 (3.8)

Data are presented as *n* (%) unless otherwise indicated. Isotonic estimated success probability was calculated using isotonic regression with the pooled-adjacent-violators algorithm (PAVA).

Quadriceps weakness was defined as an Oxford Muscle Strength Scale score < 5 at 6 h after PENG block.

Rescue analgesia refers to intravenous fentanyl administered during positioning for spinal anesthesia.

### 3.3 Secondary outcomes

Secondary efficacy and safety outcomes by concentration are summarized in Table 3. Seven patients (13.5%) required rescue analgesia with intravenous fentanyl during spinal anesthesia positioning, all of whom had failed blocks. After rescue analgesia, all seven patients successfully assumed the required lateral position and completed spinal anesthesia without further intervention; no patient required conversion to general anesthesia. The median PENG block performance time was 11 min (IQR: 10–12), with no significant difference between successful and failed block groups (Table 1).

EOSP scores showed limited dispersion, with 50 patients (96.2%) scoring two and two patients (3.8%) scoring three. Both physician and patient satisfaction scores were five in all patients. Given the absence of variability, the satisfaction scores were not informative for between-concentration comparisons and are reported descriptively only.

Among the 45 patients with successful blocks, the overall median onset time was 20.0 (17.5, 20.0) min. There was no statistically significant difference in onset time among the effective concentration groups (median onset time: 20.0 min at 0.20%, 20.0 min at 0.25%, and 17.5 min at 0.30%; *P* = 0.167, Kruskal–Wallis test).

### 3.4 Adverse events

Quadriceps weakness was observed at 6 h after PENG block in 12 patients (23.1%), and its incidence increased with increasing ropivacaine concentration (Table 3). The concentration-specific rates were 0/1 (0.0%) at 0.10%, 0/3 (0.0%) at 0.15%, 1/19 (5.3%) at 0.20%, 9/25 (36.0%) at 0.25%, and 2/4 (50.0%) at 0.30%. At 24 h after PENG block, no patients had quadriceps weakness.

The incidence of quadriceps weakness was significantly higher in the high-concentration group (≥0.25%) than in the low-concentration group (< 0.25%) (11/29 [37.9%] vs. 1/23 [4.3%], Fisher's exact *P* = 0.007), corresponding to a relative risk of 8.72 (95% CI, 1.21–62.72) and an odds ratio of 13.44 (95% CI, 1.93–303.91) (Table 2). Because quadriceps weakness was relatively common in the high-concentration group, the relative risk was considered the more clinically interpretable effect measure; the odds ratio was reported for completeness only. A significant concentration-dependent trend was also observed across increasing ropivacaine concentrations (Cochran–Armitage trend test, *P* = 0.007).

In exploratory logistic regression models with concentration entered as a continuous variable scaled per 0.05% increase, higher ropivacaine concentration was associated with increased odds of quadriceps weakness at 6 h (unadjusted OR, 4.81; 95% CI, 1.62–20.80; *P* = 0.013). This association remained significant after adjustment for age, sex, BMI, ASA physical status, and pre-block NRS score (adjusted OR, 5.56; 95% CI, 1.73–26.50; *P* = 0.011) (Table 2). The very wide confidence intervals reflect the small number of weakness events at the extreme concentration levels and should be interpreted as indicating direction rather than precise magnitude of effect.

Nausea and vomiting occurred in two patients (3.8%). No local anesthetic systemic toxicity, clinically significant hypotension, oxygen desaturation, bleeding, hematoma, or infection was observed.

## 4 Discussion

Selecting an appropriate concentration and dose of local anesthetic is important in peripheral nerve block practice because higher concentration or dose may improve block reliability but can also increase dose-related adverse effects, including unintended motor impairment. A variety of sequential methodologies have been employed in dose-finding studies for regional anesthesia, including traditional up-and-down approaches that primarily target median effective levels (21, 22, 24). However, median effective targets (e.g., ED50/EC50 or, in concentration-finding contexts, MEC50) often have limited direct clinical relevance, as clinicians typically require concentrations associated with higher success rates (e.g., ED90 or ED95) to ensure reliable analgesia. The biased-coin design (BCD) addresses this limitation by directly estimating higher quantiles of the dose-response curve, and its sequential, adaptive nature can reduce exposure to unnecessarily high concentrations while focusing allocation around the target region of the dose-response curve (20, 23).

In the present study, we employed the BCD combined with isotonic regression to estimate the effective concentration of ropivacaine for ultrasound-guided PENG block in elderly patients with femoral intertrochanteric fracture requiring positioning for spinal anesthesia. Our results demonstrated that the MEC90 was 0.25%, while the MEC50 was 0.20%. Sensitivity analyses using probit and logit dose-response models yielded broadly comparable estimates, supporting the overall consistency of the concentration-response relationship despite minor between-method differences in MEC90 estimation. Notably, quadriceps weakness at 6 h was more frequent at 0.25% than at 0.20% (36.0% vs. 5.3%). Because our safety analysis was exploratory and not powered for formal between-concentration comparisons, we do not recommend adopting 0.20% ropivacaine as a routine starting concentration on the basis of the present data. Rather, the lower weakness rate observed at 0.20% should be regarded only as a hypothesis-generating safety signal to be examined in future dose-separation studies.

This prospective biased-coin dose-finding trial provides preliminary concentration-response data for estimating the MEC90 of ropivacaine for PENG block positioning analgesia, an endpoint that has not been formally evaluated in prior prospective trials. Previous studies have typically used fixed concentrations and volumes of ropivacaine for PENG block (25–27), without formally evaluating the concentration that best balances analgesic efficacy and motor preservation. Given the single-center design, fixed-volume protocol, and clinically defined primary endpoint, these findings should be viewed as preliminary dose-finding data rather than as a definitive practice recommendation. The results may be useful mainly for designing future studies that separate concentration, volume, and total-dose effects and evaluate motor function with quantitative methods.

An important exploratory signal in our study was the concentration-dependent rise in quadriceps weakness; however, we emphasize upfront that safety comparisons across concentrations were not powered for formal confirmatory inference. This motor impairment signal merits mechanistic discussion contextualized by our uniform 20 mL injection volume and the inherent anatomical proximity of the PENG injection plane to femoral motor branches. The PENG block target plane lies between the psoas tendon and superior pubic ramus, adjacent to the femoral nerve trunk and its motor branches supplying the quadriceps muscle (17, 19, 28, 29), making unintended exposure of femoral motor branches to local anesthetic possible in some cases. In every patient we visually confirmed sub-tendinous spread in real time (Figure 1B). However, we did not quantify the extent of spread (e.g., by measuring cephalocaudal or mediolateral extension in stored ultrasound clips). The fixed 20 mL volume used across all participants may have contributed to off-target spread. Volumes exceeding the minimum required for target-plane spread could increase the likelihood of anterior spread toward the femoral nerve trunk or its motor branches. Since total ropivacaine dose rises alongside concentration under a fixed-volume protocol, the observed higher rate of quadriceps weakness at concentrations ≥0.25% cannot be definitively attributed to concentration alone; it may reflect the combined or inseparable effects of higher local anesthetic concentration and greater total drug mass. Clinically, this is of particular concern in elderly patients with hip fractures, as quadriceps weakness can elevate fall risk and delay postoperative rehabilitation (30). Importantly, the quadriceps weakness observed at 6 h had fully resolved by 24 h in all affected patients, suggesting that the motor impairment associated with higher ropivacaine concentrations was transient and consistent with the expected duration of action of ropivacaine. This transient nature mitigates, but does not eliminate the clinical concern, given the heightened fall risk in elderly patients during the early postoperative period.

Although higher concentration was associated with quadriceps weakness in exploratory analyses, these safety findings should be interpreted cautiously. The sequential dose-finding design was not powered or intended for formal safety comparisons between concentration strata; additionally, the total sample size was modest, with relatively few patients at the extreme concentration levels (0.10%, 0.15%, and 0.30%). Consequently, the estimated effect sizes for weakness (including relative risk [RR] and odds ratio [OR]) were associated with wide confidence intervals, indicating limited precision. These results support the presence of a signal toward concentration-related motor impairment but should not be interpreted as providing definitive safety thresholds for motor impairment.

The discrepancy between isotonic and parametric MEC90 estimates also merits discussion. Isotonic regression is constrained to the tested concentration levels, whereas probit and logit models interpolate between concentrations under distributional assumptions. Given the sharp increase in success rate between 0.15 and 0.20%, the model-based MEC90 estimates fell between 0.20 and 0.25%. These findings support the robustness of a narrow effective concentration range but should not be interpreted as justification for replacing the prespecified isotonic MEC90. Because this range is narrower than the practical granularity of routine ropivacaine preparation, future studies should prioritize dose-separation designs rather than direct comparison of adjacent concentrations.

The EOSP and satisfaction scores showed ceiling effects, likely because rescue fentanyl was promptly administered after failed blocks and spinal anesthesia was completed in all patients. These outcomes should therefore be regarded as descriptive rather than discriminatory measures of concentration-specific efficacy. Consequently, the primary outcome should be interpreted as a clinically meaningful analgesic endpoint rather than an anatomical confirmation of successful capsular sensory blockade.

Previous studies have suggested that PENG block may provide favorable positioning analgesia compared with FNB and FICB in selected hip surgery settings (31, 32), while also potentially offering anatomical and clinical advantages over fascia iliaca-based approaches—particularly in terms of coverage of the articular branches innervating the anterior hip capsule, which are critical for mediating hip fracture-related pain (33). Accumulating evidence supports the analgesic value of peripheral nerve blocks in the perioperative management of hip fractures, and recent comparative studies have further suggested the potential for better motor preservation with PENG block than with FNB (34). Consistent with these findings, Lin et al. (35) reported a significantly higher rate of preserved quadriceps muscle strength in patients receiving PENG block compared with those receiving FNB. Similarly, Allard et al. (36) assessed quadriceps muscle strength using the Medical Research Council (MRC) scale and found a median MRC score of 5 (interquartile range [IQR], 4–5) in the PENG group, compared with a median score of 2 (IQR, 2–3.8) in the FNB group. The proposed anatomical basis for this motor-sparing potential is that PENG block is intended to target sensory articular branches of the anterior hip capsule rather than the femoral motor branches controlling quadriceps function (16, 37). Our findings extend this literature by suggesting that motor impairment may also depend on concentration or total dose when a fixed 20 mL volume is used.

To contextualize our findings within the existing literature, we compared our results with published data on positioning analgesic success rates and quadriceps weakness incidence. Regarding positioning analgesic efficacy, our results appear broadly consistent with published studies evaluating PENG block for hip fracture positioning pain; in contrast, FNB and FICB have been associated with slightly lower positioning analgesic success rates (36, 38), suggesting that PENG block may be a useful analgesic option in this setting. Quadriceps weakness incidence is consistent with the broader literature on PENG block, which reports quadriceps weakness rates ranging from 0 to 45% (29). Because the injection volume was fixed at 20 mL, the observed weakness gradient may reflect concentration, total dose, spread pattern, or a combination of these factors. A direct 0.20%-vs.−0.25% head-to-head comparison would have limited practical value because the resolution falls below the routine granularity of ropivacaine stocking and compounding. Future confirmatory research should instead prioritize a factorial concentration × volume (dose-separation) design, for example testing whether a reduced volume (13–15 mL) at an effective concentration preserves analgesia while reducing the motor-weakness gradient; and trials powered on quantitative motor endpoints (handheld dynamometry) together with clinically meaningful functional outcomes such as time-to-mobilization and in-hospital fall incidence, rather than the semiquantitative Oxford scale.

## 5 Limitations

Several limitations should be acknowledged. First, block success was defined using a clinical pain endpoint, namely NRS ≤ 3 at rest and during passive leg raise, rather than objective confirmation that the anterior hip capsular articular branches were blocked. Pain scores may be influenced by anxiolysis, spontaneous fluctuation of fracture pain, partial spread, or systemic absorption. This limitation was compounded by ceiling effects in EOSP and satisfaction scores after rescue fentanyl, which limited their value as independent corroborative measures. Because the PENG block targets deep articular branches without a consistent cutaneous sensory distribution, direct bedside confirmation is challenging. Future studies should incorporate predefined corroborating assessments, such as structured sensory testing where feasible, quantitative sensory testing, standardized passive-movement pain evaluation, and quantitative or recorded ultrasound documentation of injectate spread. Second, the BCD resulted in unequal sample sizes across concentration levels, with few observations at the lowest and highest tested concentrations, limiting precision at the extremes. Third, this was a single-center study, and all blocks were performed by one experienced anesthesiologist; therefore, the findings may not directly transfer to less experienced operators or other practice settings. Fourth, the fixed 20 mL volume precluded separation of concentration, volume, and total-dose effects, and ultrasound spread was confirmed qualitatively rather than quantified on stored images. Fifth, quadriceps weakness was assessed with the semiquantitative Oxford Muscle Strength Scale rather than quantitative dynamometry. Sixth, although intraoperative anesthesia depth and rescue analgesic interventions administered after the 30-min primary endpoint assessment could not alter the binary block-success outcome, these perioperative variables may influence later postoperative secondary assessments including quadriceps weakness and early mobilization capacity. We standardized preoperative sedation and adjusted for selected baseline covariates, including age, sex, BMI, ASA physical status, and pre-block NRS score, in exploratory logistic regression models. However, unmeasured potential confounders, such as chronic long-term analgesic exposure, precise timing of preoperative analgesic administration, and postoperative mobilization protocols, were not incorporated into statistical adjustment. Therefore, residual confounding cannot be excluded. Because this sequential biased-coin dose-finding study was designed primarily to estimate the MEC90 rather than to perform covariate-adjusted safety inference, the adjusted regression model should be interpreted as exploratory. Finally, the study was not powered to detect rare adverse events, and longer-term outcomes such as postoperative delirium, hospital length of stay, and 30-day mortality were not assessed.

## 6 Conclusions

In elderly patients with femoral intertrochanteric fracture receiving ultrasound-guided PENG block with a fixed 20 mL volume of ropivacaine, the prespecified isotonic regression-estimated MEC90 for relieving spinal anesthesia positioning pain was 0.25%. Exploratory safety analyses suggested that quadriceps weakness at 6 h was more common at concentrations ≥0.25%, but these analyses were underpowered and hypothesis-generating. Future studies should determine whether modifying volume, concentration, or total dose can preserve analgesia while reducing motor impairment.

## Data Availability

The original contributions presented in the study are included in the article/Supplementary material, further inquiries can be directed to the corresponding author.

## Supplementary material

Supplementary Figure S1 Bootstrap distributions of MEC50 and MEC90. (A) The bootstrap distribution of MEC50 is concentrated at 0.20%, reflecting limited variation in the isotonic estimate across 10,000 resamples. (B) The bootstrap distribution of MEC90 is centered at 0.25%, with additional resampled estimates at adjacent tested concentrations, indicating greater uncertainty in the upper-tail estimate due to the discrete concentration levels and limited sample size at the extremes.
